# Hemodynamic Relevance Evaluation of Coronary Artery Anomaly During Stress Using FFR/IVUS in an Artificial Twin

**DOI:** 10.1016/j.jaccas.2024.102729

**Published:** 2024-12-04

**Authors:** Joël Illi, Anselm W. Stark, Marc Ilic, Dani Soares Loureiro, Dominik Obrist, Isaac Shiri, Lorenz Räber, Andreas Haeberlin, Christoph Gräni

**Affiliations:** a Department of Cardiology, Inselspital, Bern University Hospital, University of Bern, Bern, Switzerland; b ARTORG Center for Biomedical Engineering Research, University of Bern, Switzerland; c Translational Imaging Center, Sitem Center, University of Bern, Switzerland

**Keywords:** 3D-printed patient-specific phantom, 3DPSP, AAOCA, ACAOS, additive manufacturing, AM, anomalous aortic origin of a coronary artery, artificial twin, CCTA, coronary artery anomaly, dobutamine, FFR, fractional flow reserve, hemodynamic testing, intravascular ultrasound, IVUS

## Abstract

Anomalous aortic origin of coronary artery can lead to ischemia. Due to the limitations of invasive catheterization dobutamine stress testing, an alternative noninvasive approach is desired. A 65-year-old woman with atypical chest pain was referred for coronary computed tomography angiography. Although coronary artery disease was excluded, a right anomalous aortic origin of coronary artery with an interarterial and intramural course was discovered. The patient underwent invasive coronary angiography with a dobutamine stress test, which revealed a pathologic fractional flow reserve (ie, dobutamine fractional flow reserve) of 0.76 (normal >0.8) and lateral ostial compression in dobutamine intravascular ultrasound. A physical replication, using a patient-specific 3-dimensional–printed phantom was created based on coronary computed tomography angiography and evaluated in a flow loop under the same hemodynamic rest and stress conditions. The 3-dimensional–printed phantom fractional flow reserve was similar with 0.78, and dobutamine intravascular ultrasound showed comparable lateral compression.

## Visual Summary

**Figure:**
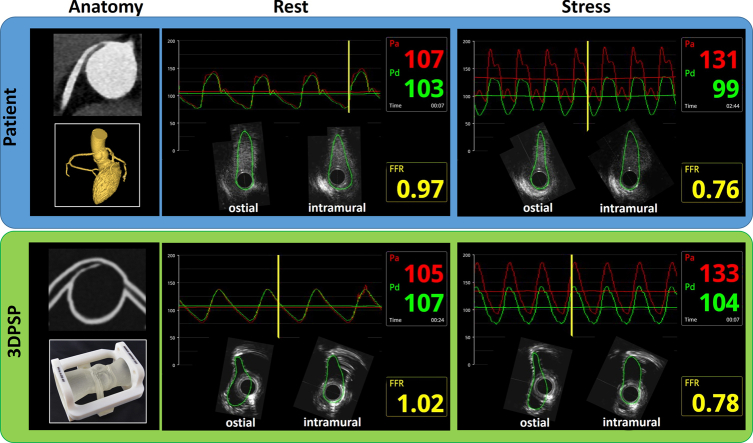

**Visual Summary:**
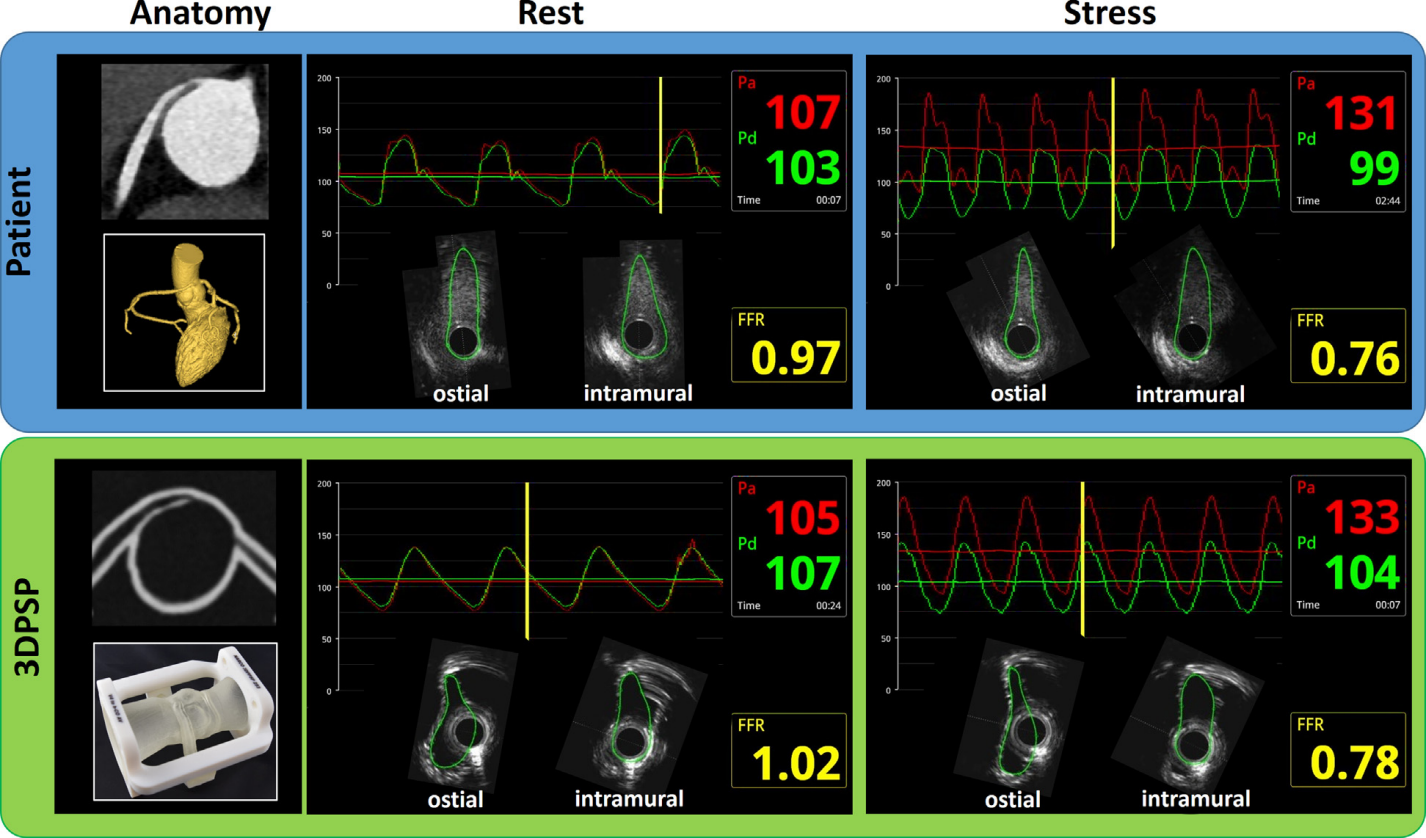
Comparison of Clinical and Artificial Twin Hemodynamics and Morphologic Changes: Fractional Flow Reserve and Intravascular Ultrasound of Anomalous Aortic Origin of Coronary Artery Under Rest and Stress Conditions The left column displays, from top to bottom, the patient’s CCTA, the patient’s 3-dimensional reconstruction, the coronary computed tomography angiography scan of the 3DPSP, and an image of the 3DPSP. The center column shows, from top to bottom, the patient’s resting measurements, including FFR and IVUS for both the ostial and intramural regions, followed by the 3DPSP’s resting measurements for FFR and IVUS in the same regions. The right column presents, from top to bottom, the patient’s stress measurements for FFR and IVUS (ostial and intramural, i.e. morphologic changes), and the 3DPSP’s corresponding stress measurements. 3DPSP = 3-dimensional printed patient-specific phantom; CCTA = coronary computed tomography angiography; FFR = fractional flow reserve; IVUS = intravascular ultrasound.

**Figure 1:**
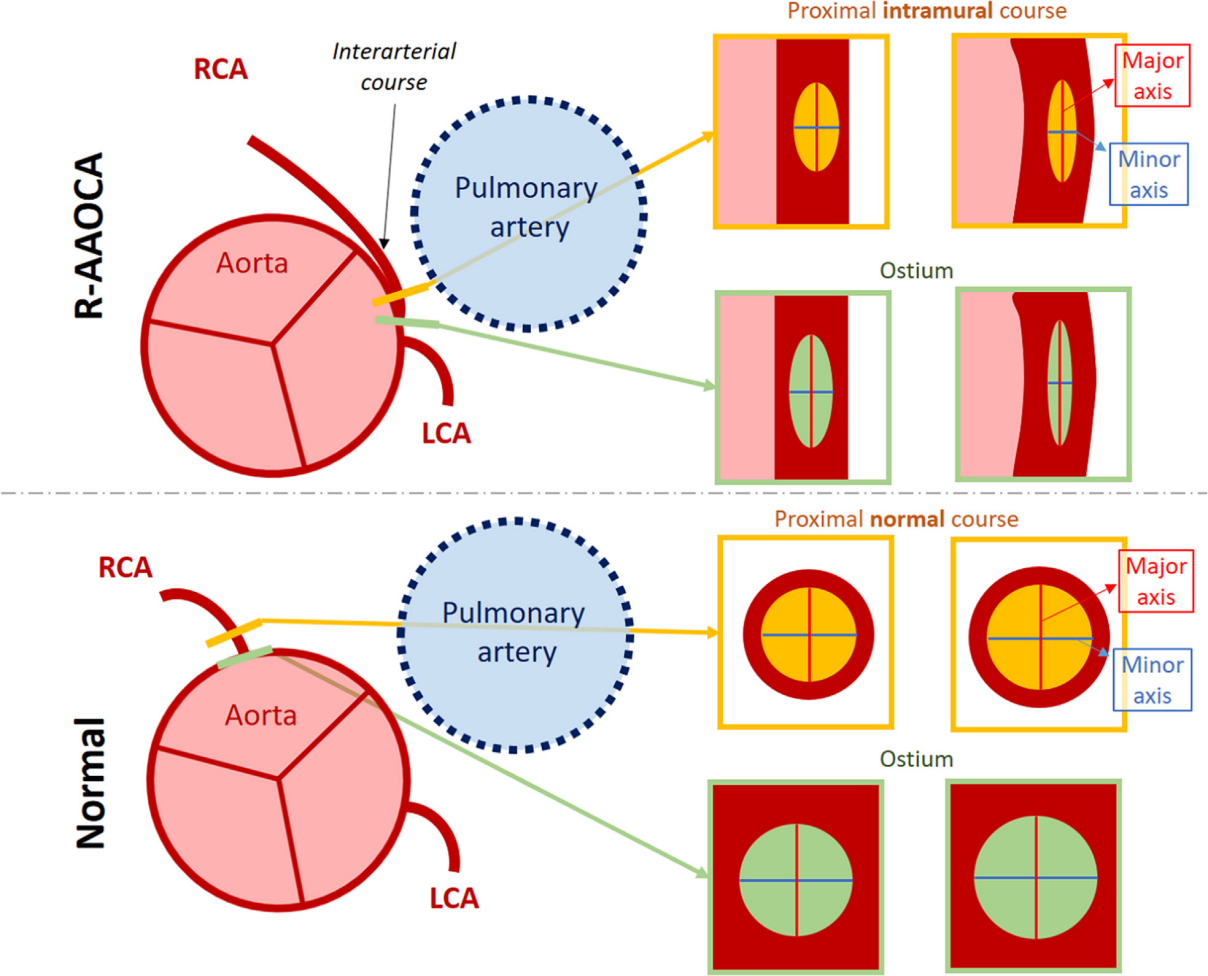
Schematic Overview of the Normal Coronary Artery Anatomy and Anomalous Aortic Origin of Coronary Artery and Depiction of the Anatomic Geometric Changes in Rest and Stress Conditions (Top) R-AAOCA with an interarterial course between the aorta and the pulmonary artery and intramural course (within the tunica media of the aortic wall). The green markers on the top panel indicate the ostium of the anomalous vessel with the slit-like vessel shape, whereas the orange markers indicate the locations of the intramural vessel lumen. The elliptic ratio is represented by the major axis (red) divided by the minor axis (blue). During stress, both the ostial and intramural stenosis are accentuated (decrease in lumen area), whereas the elliptic ratio increases. (Bottom) Normal anatomy is shown as a reference. Here, the ostium and the normal proximal vessel course are round with equal lengths of the major and minor axes. During stress, the lumen area may slightly increase, whereas the elliptic ratio remains unchanged. LCA = left coronary artery; R-AAOCA = right anomalous aortic origin of coronary artery; RCA = right coronary artery.

Anomalous aortic origins of coronary arteries (AAOCAs) with an interarterial/intramural course are associated with a higher risk of myocardial ischemia and sudden cardiac death.^1^^,^^2^ The frequent presence of a slit-like ostium and the oval vessel shape of the intramural course (within the tunica media of the aortic wall) in AAOCA with an interarterial course differs from the round ostium and proximal vessel in normal anatomy (Figure 1). Under stress conditions, an accentuation of ostial and intramural stenosis (decrease in lumen area) and an increase in the elliptic ratio, induced by unidirectional lateral compression from the aorta, can potentially lead to significant hemodynamic changes.^1^^,^^3^ Due to the complex pathomechanism, involving both fixed and dynamic components in AAOCA, the proposed gold standard to assess hemodynamic relevance is by invasive measured fractional flow reserve (FFR) and intravascular ultrasound (IVUS) during mimicked exercise conditions by dobutamine-atropine-volume challenge. However, invasive catheterization at maximal heart rate is uncomfortable for patients, requires expertise, is costly, and carries the very rare risk of dissection and failure to intubate the catheter into the anomalous ostium. Therefore, an alternative noninvasive method to visualize coronary arteries at rest and under stress, including vessel-based ischemia assessment, is desired. Recently, 3-dimensional printing has emerged as a promising tool in medicine^4^; however, its clinical use is still in early stages.^5^ Few studies have assessed its accuracy, its applicability,^6^ and the hemodynamic properties of AAOCA models.^7^^,^^8^ Our aim was to assess the feasibility of creating an artificial twin by using a 3-dimensional–printed patient-specific phantom (3DPSP) based on noninvasive coronary computed tomography angiography (CCTA) in a flow loop setup, and physically replicate the patient’s specific hemodynamic rest- and stress conditions and compare them to the clinical evaluation.

**Take-Home Message**

• This artificial twin prototype, using a 3-dimensional printed phantom, enables external hemodynamic stress testing in a flow-loop setup, demonstrating significant potential for clinical evaluation of anomalous aortic origin of the coronary artery.

## Case Summary

A 65-year-old woman with atypical chest pain was referred for CCTA. Although coronary artery disease was excluded, a right AAOCA with an interarterial and intramural course and right coronary dominance was discovered. The patient underwent coronary angiography to assess hemodynamic relevance during a dobutamine-atropine-volume challenge. The heart rate increased from 80 to 144 beats/min and aortic pressure increased from 145/78 to 188/89 mm Hg (Table 1) under stress. The pressure ratio between the aorta and distal to the stenotic segment was measured at rest (0.97) and fractional flow reserve during dobutamine-atropine-volume challenge (FFR_dobutamine_ = 0.76) (Table 2). IVUS automated pullback was performed at rest and under dobutamine, from the distal right coronary artery (RCA) through the intramural segment to the aorta. Ostial and intramural minimal lumen area, elliptic ratio, and stenosis were then evaluated. The ostial lumen area from rest to stress decreased from 3.5 to 2.6 mm^2^, whereas the elliptic ratio increased from 4 to 6, demonstrating a dynamic component with lateral compression (Table 3, Videos 1 and 2). After evaluation, a shared decision favored a conservative approach over surgery due to borderline results, mild symptoms, and the patient’s noncompetitive sports lifestyle. A beta-blocker was prescribed, competitive sports were restricted, and no cardiovascular events occurred at the 6-month follow-up. After the patient’s treatment and follow-up, this case was retrospectively selected to test our noninvasive artificial twin approach as a proof of concept; hence, there was no impact or additional risk to the patient.

**Table 1:** Flow Loop Parameters Used to Mimic the Patient’s Hemodynamic Conditions

Origin	Parameter	Rest	Stress
Patient data	Heart rate, beats/min	80	144
	Aorta systolic pressure, mm Hg	142	187
	Aorta diastolic pressure, mm Hg	77	92
Literature	Cardiac output, L/min	5	15
	Right coronary artery flow, mL/min	110	220

**Table 2:** Comparison of the Hemodynamic Parameters and Resulting Measurements of the Patient and the 3DPSP for Rest and Stress Conditions

Results From Clinical Evaluation	Patient	3DPSP	Patient	3DPSP
	Rest		Stress	
Heart rate, beats/min	80		144	
Fractional flow reserve	0.97	1.02	0.76	0.78
Aorta systolic pressure, mm Hg	145	139	188	187
Aorta mean pressure, mm Hg	107	105	131	133
Aorta diastolic pressure, mm Hg	78	77	89	91
Distal systolic pressure, mm Hg	140	138	133	143
Distal mean pressure, mm Hg	103	107	99	104
Distal diastolic pressure, mm Hg	76	80	63	73
Ostial lumen area, IVUS, mm^2^	3.51	3.06	2.59	2.71
Ostial elliptic ratio, IVUS	3.97	2.68	5.98	4.34
Ostial major axis, IVUS, mm	3.93	3.26	3.81	3.72
Ostial minor axis, IVUS, mm	0.99	1.22	0.64	0.86
Ostial lateral compression, IVUS, %			26	13
Intramural lumen area, IVUS, mm^2^	3.60	3.03	3.46	3.16
Intramural elliptic ratio, IVUS	3.08	2.76	3.74	2.88
Intramural major axis, IVUS, mm	3.59	3.13	3.87	3.13
Intramural minor axis, IVUS, mm	1.16	1.14	1.04	1.08
Intramural lateral compression, IVUS, %			∼0	∼0

3DPSP = 3-dimensional–printed patient-specific phantom; IVUS = intravascular ultrasound.

**Table 3:** Comparison of CT and IVUS Rest Measurements for the Patient and the 3DPSP

	Patient		3DPSP	
	IVUS Rest	CT	IVUS Rest	CT
Ostial lumen area, mm^2^	3.51	3.45	3.06	3.47
Ostial elliptic ratio	3.97	3.25	2.68	3.08
Ostial major axis, mm	3.93	3.90	3.26	3.70
Ostial minor axis, mm	0.99	1.20	1.22	1.20
Intramural lumen area, mm^2^	3.60	3.54	3.03	3.64
Intramural elliptic ratio	3.08	2.77	2.76	2.54
Intramural major axis, mm	3.59	3.60	3.13	3.30
Intramural minor axis, mm	1.16	1.30	1.14	1.30

3DPSP = 3-dimensional–printed patient-specific phantom; CT = computed tomography; IVUS = intravascular ultrasound.

## Procedural Steps

**Figure 2:**
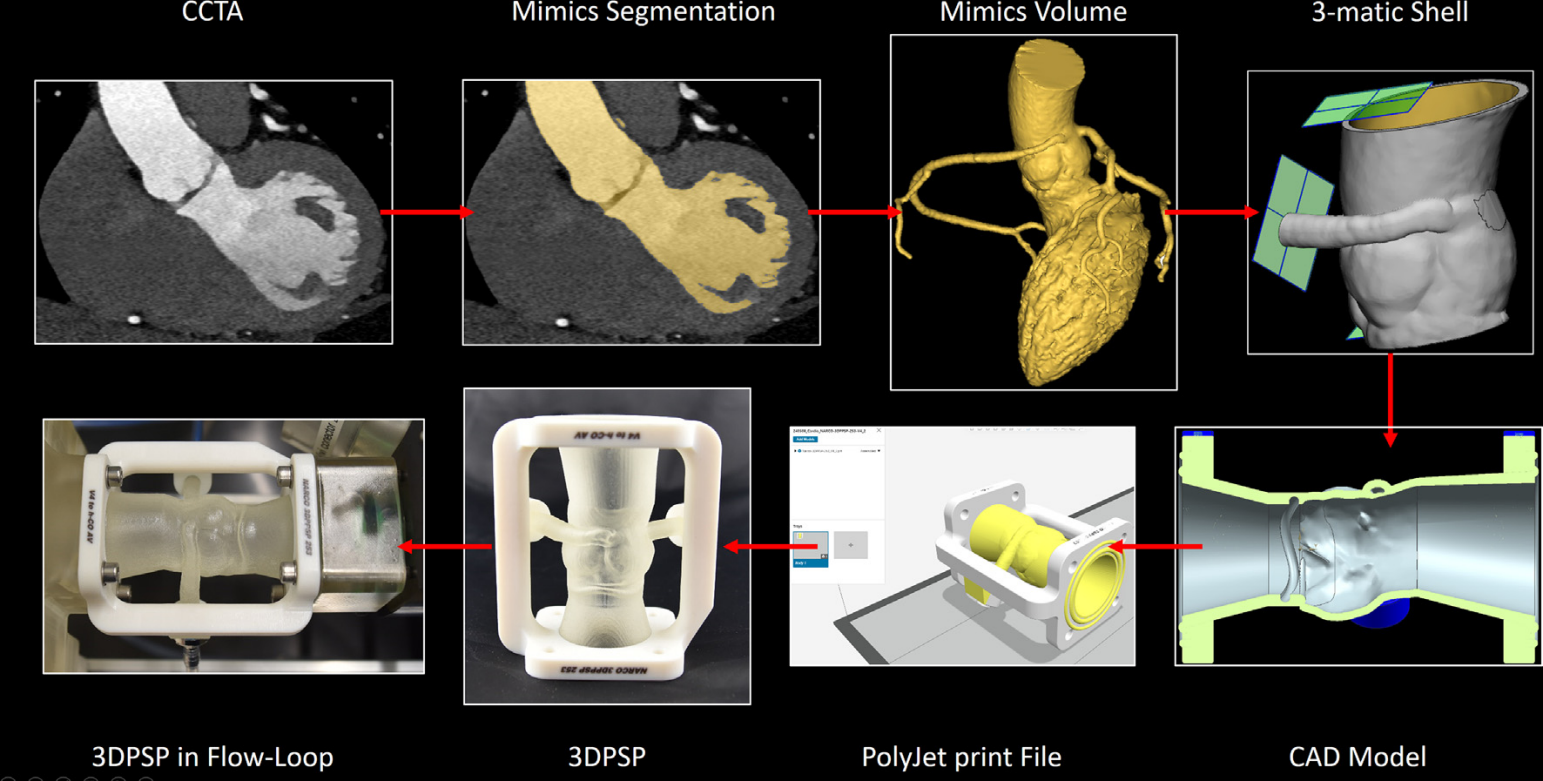
Workflow of 3DPSP Creation Workflow from CCTA-based creation of the 3DPSP to its integration into hemodynamic testing in the flow loop. 3DPSP = 3-dimensional–printed patient-specific phantom; CAD = computer-aided design; CCTA = coronary computed tomography angiography.

A 3DPSP was created from CCTA to be tested in a flow loop under patient-specific conditions. The workflow steps were as follows (Figure 2).

### Phantom manufacturing

**Figure 3:**
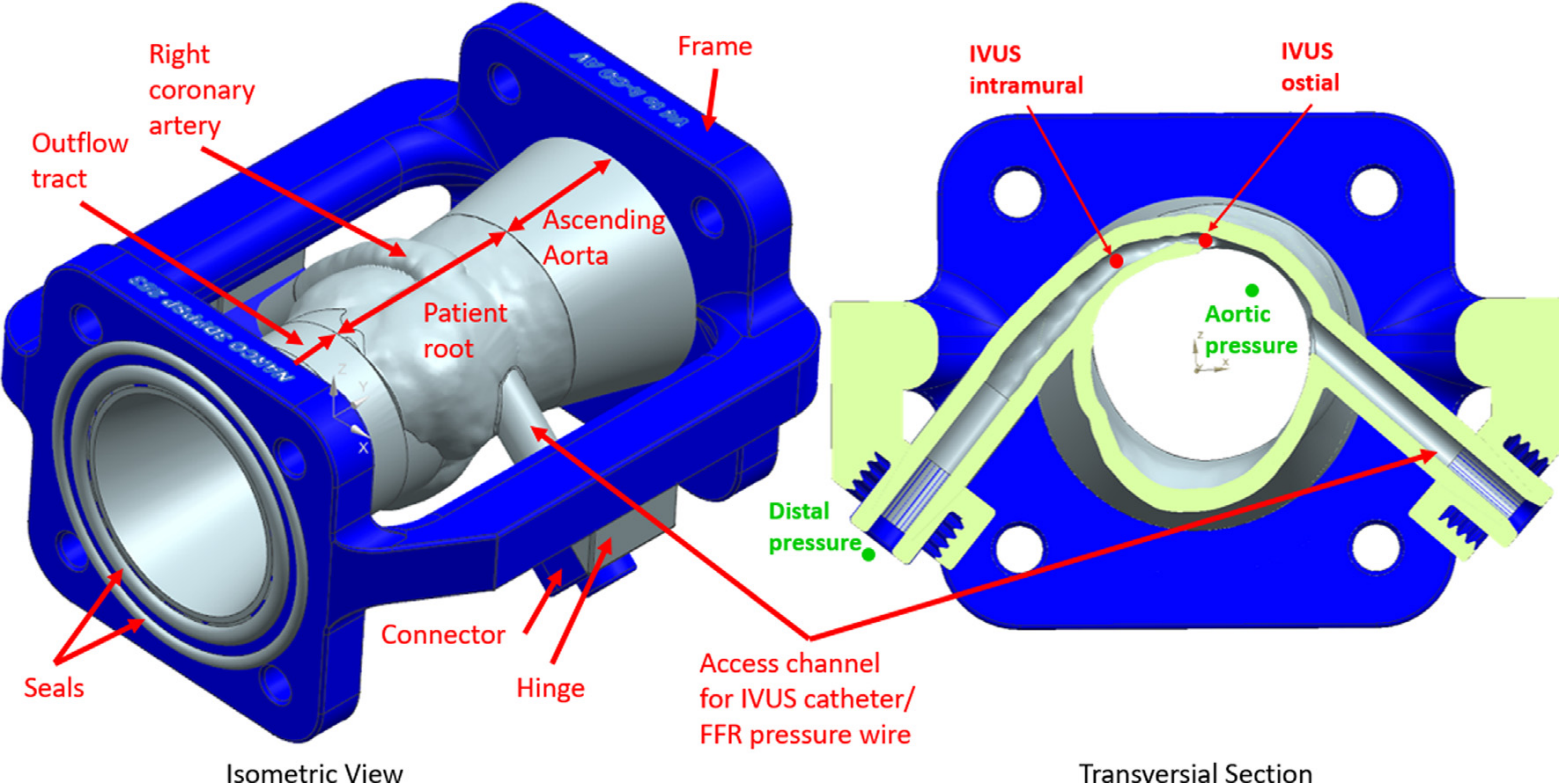
Detailed 3-Dimensional–Printed Patient-Specific Phantom Computer-Aided Design In the left panel, the isometric view of the 3-dimensional–printed patient-specific phantom (3DPSP) with marked components is shown. The connectors were used to provide access to the phantom with the clinical instrumentation through the access channel and to connect the right coronary artery to the coronary circuit. The connectors were fixed to the frame via flexible hinges to prevent the vessels from kinking. The hinges were made of flexible material to allow for limited movement of the right coronary artery, channel, and aorta during the cardiac cycle and reduce stresses at the contact points. In the right panel, the transversal section through the 3DPSP at the center of the right coronary artery ostium on the right is shown, with marked locations for the clinical pressure measurements in green and the locations for the cross-sectional evaluation of the intravascular ultrasound recording in red. The light green surface marks the cutting surface of the section. Blue represents VeroPureWhite (Stratasys) parts (frame and connectors). Gray represents Agilus30Clear (Stratasys) parts (seals, anatomy and hinges). FFR = fractional flow reserve; IVUS = intravascular ultrasound.

First, the patient’s mid-diastolic–acquired CCTA images were segmented using Mimics 25.0 (Materialise) (Figure 3). The 3-matic 17.0 (Materialise) was used for processing, and the model was imported into NX 1884 (Siemens PLM Software) to design connections to the flow loop, including the artificial left ventricular outflow tract and ascending aorta. The model was then imported into GrabCAD Print 1.86 (GrabCAD) to assign materials and create the print file for additive manufacturing. For the anatomy, hinges, and seals, Agilus30Clear (Stratasys) was selected based on a study performed by our group and other studies.^5^^,^^7^^,^^8^ For the rigid frame and connectors, VeroPureWhite (Stratasys) was used, together with support SUP706B (Stratasys). The phantom was then printed on a Stratasys J750 PolyJet printer and cleaned with a mixture of waterjet brushing and rinsing with a 2% sodium hydroxide solution.

Table 3 compares the patient’s CCTA measurements with the CT-scanned 3DPSP.

### Flow loop setup

**Figure 4:**
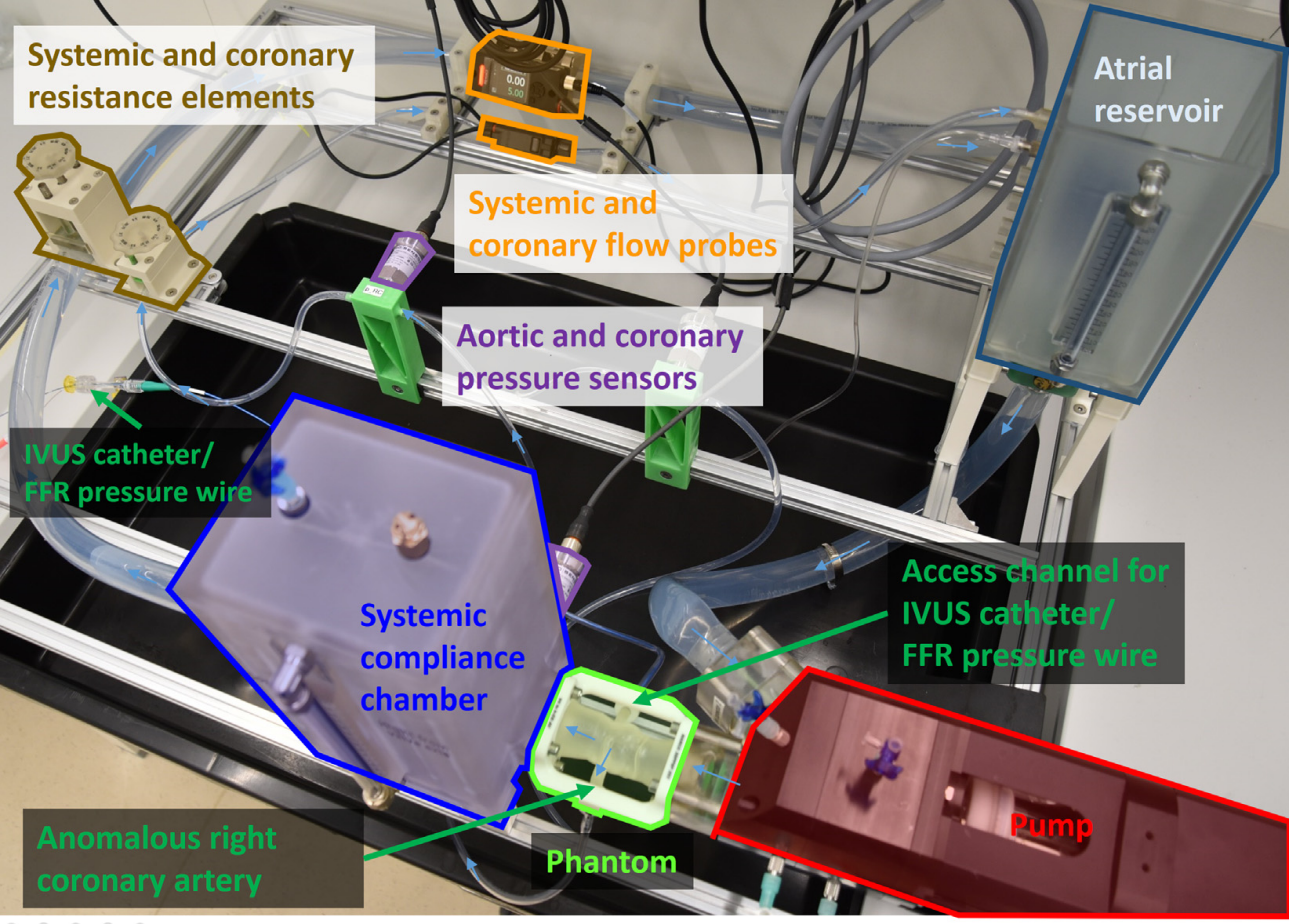
Flow Loop Setup Flow loop setup with the ability to assess FFR and IVUS anatomic deformation of the anomalous coronary artery in resting and stress conditions (mimicked physical exercise with increasing heart rate and blood pressure). FFR = fractional flow reserve; IVUS = intravascular ultrasound.

The custom-made flow loop allowed hemodynamic testing under stress, with control over heart rate, cardiac output, aortic blood pressure, compliance, and peripheral resistance. The setup consists of the ViVitro SuperPump AR Series and PumpHead (ViVitro Labs) for the left ventricle, an aortic valve, the aortic root phantom, a compliance chamber, systemic resistance element, left atrium reservoir, and a mitral valve (Figure 4). The main circuit is created by transparent polyvinyl chloride tubing with an internal diameter of 25 mm. In parallel, there is a secondary circuit with transparent polyvinyl chloride tubing with 4-mm internal diameter, and a resistance element, connecting the RCA to the left atrium reservoir. Both circuits are instrumented with ultrasound-based flow probes and pressure sensors.

### Hemodynamic assessment of the phantom in the flow loop setup

#### Preparation

A 6-F guide catheter was inserted into the phantom’s sinus portion, via the access channel. The wireless pressure wire was advanced into the ostium through the guide catheter, with the sensor portion connected to the FFR console. The IVUS catheter was then threaded onto the pressure wire and advanced behind the pressure sensor. The instrumentation used was identical to the clinical evaluation (Table 4).

**Table 4:** Equipment List With Materials and Instrumentation for the 3DPSP, the Flow Loop Setup, and the Clinical Evaluation

Phantom	
Patient-specific structure	2 mm, Agilus30Clear
Inlet	3 mm, Agilus30Clear
Outlet	2-3 mm, Agilus30Clear
RCA, access channel	2 mm, Agilus30Clear
Connectors	G1/4, VeroPureWhite
O-ring seals	2 mm, Agilus30Clear
Connector hinges	Agilus30Clear
Contact plates and frame	8 mm, VeroPureWhite
Flow Loop	
Chambers, connectors, resistance elements, and valves	Custom, Anycubic Basic Clear Resin
Systemic tubing	25 × 33 mm transparent polyvinyl chloride tubing
Coronary tubing	4 × 7 mm transparent polyvinyl chloride tubing
Pump and pump head	ViVItro SuperPump AR Series
Pressure sensors	BD-Sensors DCT 533
Flow probes	Keyence FD-H,FD-X
DAQ	Keyence NQ-MP8L
Clinical Instrumentation	
Aortic pressure transducer	Smiths Medical LogiCal
Distal pressure wire	St. Jude Medical PressureWire X
FFR console	St. Jude Medical LightLab console
IVUS catheter	Boston Scientific OptiCross18 IVUS catheter
IVUS console	Boston Scientific AVVIGO
Guide catheter	6-F, straight, shortened

DAQ = data acquisitioning; FFR = fractional flow reserve; IVUS = intravascular ultrasound; RCA = right coronary artery.

#### Replicating the patient-specific hemodynamic conditions

Table 1 shows the parameters used on the flow loop to mimic the patient-specific conditions of rest and stress described previously. The cardiac output (5 L/min) and RCA flow (110 mL/min)^9^ for the rest condition were based on literature data. For the stress condition, the cardiac output was set to 15 L/min and the RCA flow to 220 mL/min, based on clinical experience and comparable to data from literature.^7^^,^^10^

#### Experimental evaluation of the 3DPSP

FFR and IVUS measurements were performed as during the patient’s clinical evaluation. Figure 3 shows a cross section of the 3DPSP through the center of the anomalous right coronary ostium, with marked locations of the pressure recordings and the IVUS imaging slices that were evaluated. In the 3DPSP, the patient-specific rest and stress conditions could be closely reproduced, rendering an accurate representation of cardiac stress. Furthermore, the 3DPSP (Figure 2, Figure 3, Figure 4) and setup were able to achieve high cardiac output of 15 L/min and an anomalous right coronary flow of 220 mL/min and aortic pressures during stress conditions (3DPSP: 187/91 mm Hg, patient: 188/89 mm Hg).

Clinical FFR_dobutamine_ and FFR in 3DPSP were similar (0.76 vs 0.78, respectively) with both showing a borderline hemodynamically relevant right AAOCA (cutoff FFR ≤0.8). Ostial and intramural areas were compared between the patient and 3DPSP. The patient had a slightly larger ostial area (3.51 vs 3.06 mm^2^) and higher elliptic ratio (3.97 vs 2.68) at rest. Both showed lateral compression during stress, with the area decreasing to 2.59 mm^2^ for the patient and 2.71 mm^2^ for the 3DPSP. The patient’s compression was greater (26% vs 13%) (Videos 3 and 4). Values, FFR pressure curves, and IVUS images are shown in Table 2 and Figure 3.

## Discussion

There are limited data on the use of 3DPSP in the clinical setting of AAOCA. Hatoum et al^7^ were among the first to propose using a flow loop for hemodynamic assessment of AAOCA, but they did not compare 3DPSP results to invasive measurements, limiting the reproduction of patient-specific conditions in their model. In contrast to their approach, we demonstrated morphologic changes with IVUS imaging and accurately reproduced the patient’s stress conditions using dobutamine, by adjusting aortic pressure, heart rate, cardiac output, RCA flow, and systolic ratio. Although the phantom’s RCA compression during IVUS closely mirrored the patient’s, the extent of compression was lower in the phantom. This difference may be due to the stiffer artificial wall material or the larger impression left by the IVUS catheter in the 3DPSP, possibly from a different engagement angle. However, the recorded pressure curves closely mimic the patient’s, showing similar FFR values at rest and under stress, with comparable mean pressures, despite slight differences in curve shape. The morphologic accuracy of the 3DPSP was derived from CCTA images, which have lower resolution compared to IVUS images (0.4 mm in-plane vs 0.1 mm). The effects of different materials and physiological conditions on measurements should be assessed, incorporating additional patient-specific parameters (eg, cardiac output, RCA flow, tissue fiber recruitment, dynamic peripheral resistance changes) across varying heart rates and blood pressures (see Supplemental Table 1 for current limitations).

## Conclusions

To our knowledge, for the first time, we created an artificial twin using a 3DPSP based on CCTA, enabling external hemodynamic stress testing in a flow loop setup using FFR and IVUS in AAOCA. We compared invasive measurements obtained under both resting and stress conditions, and the in vitro physical replicated physiological condition results closely matched the in vivo assessments. Given the limitations of invasive measurements, this noninvasive prototype approach holds significant potential for the clinical evaluation of AAOCA.

## Funding Support and Author Disclosures

This work was supported by the Swiss National Science Foundation (grant number 200871) to Dr Gräni. Dr Gräni has received funding from InnoSuisse, Center for Artificial Intelligence in Medicine University Bern, GAMBIT Foundation, Novartis Foundation for Medical-Biological Research, and Swiss Heart Foundation, outside of the submitted work; and serves as editor-in-chief of *The International Journal of Cardiovascular Imaging* (Springer). All other authors have reported that they have no relationships relevant to the contents of this paper to disclose.

## Appendix

Video 1 Intravascular Ultrasound Rest Patient

Video 2 Intravascular Ultrasound Stress Patient

Video 3 Intravascular Ultrasound Rest 3-Dimensional–Printed Patient-Specific Phantom

Video 4 Intravascular Ultrasound Stress 3-Dimensional–Printed Patient-Specific Phantom

## References

[1] Bigler M.R., Ashraf A., Seiler C. (2020). Hemodynamic relevance of anomalous coronary arteries originating from the opposite sinus of valsalva-in search of the evidence. Front Cardiovas Med.

[2] Grani C., Kaufmann P.A., Windecker S., Buechel R.R. (2019). Diagnosis and management of anomalous coronary arteries with a malignant course. Interv Cardiol.

[3] Schütze J., Stark A.W., Bigler M.R., Räber L., Gräni C. (2024). Misconception of 'malignant' and 'scissor-like compression' of interarterial course in anomalous aortic origin of a coronary artery: a case series. Eur Heart J Case Rep.

[4] Bernhard B., Illi J., Gloeckler M. (2022). Imaging-based, patient-specific three-dimensional printing to plan, train, and guide cardiovascular interventions: a systematic review and meta-analysis. Heart Lung Circ.

[5] Illi J., Bernhard B., Nguyen C. (2022). Translating imaging into 3D printed cardiovascular phantoms: a systematic review of applications, technologies, and validation. JACC Basic Transl Sci..

[6] Parthasarathy J., Hatoum H., Flemister D.C. (2021). Assessment of transfer of morphological characteristics of anomalous aortic origin of a coronary artery from imaging to patient specific 3D printed models: a feasibility study. Comput Methods Programs Biomed.

[7] Hatoum H., Krishnamurthy R., Parthasarathy J. (2022). Flow dynamics in anomalous aortic origin of a coronary artery in children: importance of the intramural segment. Semin Thorac Cardiovasc Surg.

[8] Illi J., Ilic M., Stark A.W. (2023). Mechanical testing and comparison of porcine tissue, silicones and 3D-printed materials for cardiovascular phantoms. Front Bioeng Biotechnol.

[9] Sakamoto S., Takahashi S., Coskun A.U. (2013). Relation of distribution of coronary blood flow volume to coronary artery dominance. Am J Cardiol.

[10] Sankaran S., Esmaily Moghadam M., Kahn A.M., Tseng E.E., Guccione J.M., Marsden A.L. (2012). Patient-specific multiscale modeling of blood flow for coronary artery bypass graft surgery. Ann Biomed Eng.

