# Supplementary material for: Hemodynamic Relevance Evaluation of Coronary Artery Anomaly During Stress Using FFR/IVUS in an Artificial Twin
Source: JACC Case Rep. 2024 Dec 4;30(1):102729. doi: 10.1016/j.jaccas.2024.102729 (PMC11733583; doi:10.1016/j.jaccas.2024.102729)
Supplement: Supplemental Table 1 [file mmc5.docx]

Supplemental Table 1 Limitations Associated With This Case and Its Explanations

| **Limitations** | **Explanation** |
| --- | --- |
| Single case study | Because only 1 case was represented, this did not provide sufficient validation of the current workflow and primarily served as a proof of concept. Further studies with larger case numbers are needed. |
| Differences observed in the ostium between the invasive measurements and the 3DPSP | The IVUS images suggest a greater influence of the IVUS catheter on the vessel wall in the 3DPSP. This may potentially be explained by a different angle of engagement compared to the invasively used catheter (JL 4.0 6.5-F Sheathless), and different tissue behavior. |
| Considerations for various designs and tissue modifications | Although Agilus30Clear is currently considered the most physiological surrogate for aortic tissue based on existing literature, it does not fully replicate physiological behavior. |
| Nonlinear behavior exhibited by aortic tissue, due to fiber recruitment mechanism, cannot be reproduced, which could particularly affect stress results | Voxel-based printing with mimicking fiber orientation may offer a promising avenue for future research to replicate varying fiber recruitment properties within vessel walls and incorporate diverse materials to account for calcifications in the aorta or coronary vessels (eg, atherosclerotic calcifications and stenosis in the case of concomitant presence of coronary artery disease). |
| Other factors which influence pathophysiological mechanism in AAOCA | The full pathophysiological mechanism in AAOCA has not yet been sufficiently investigated, and other anatomic factors, such as the wall thickness between the aorta and coronary artery, may also influence the degree of compression. |
| Influence of phasic compression | To better replicate phasic compression in systole vs diastole of the intramural segment, future research should include this additional element to simulate the phase shift in coronary flow (systole vs diastole), which was not assessed in this study. |
| Impact of wall stress and peripheral coronary resistance on the results | Incorporating myocardial wall stress and dynamic coronary peripheral resistance, which reflect changes in coronary flow during systole and diastole at varying heart rates, will be necessary, especially considering the differences between the left coronary arteries (thicker left ventricular myocardium) and the right coronary artery (thinner right ventricular myocardium). |

3DPSP = 3-dimensional–printed patient-specific phantom; AAOCA = anomalous aortic origin of coronary artery; IVUS = intravascular ultrasound.
